# On the Mechanical, Thermal, and Rheological Properties of Polyethylene/Ultra-High Molecular Weight Polypropylene Blends

**DOI:** 10.3390/polym15214236

**Published:** 2023-10-26

**Authors:** Vishal Gavande, Mingi Jeong, Won-Ki Lee

**Affiliations:** Division of Polymer Engineering, Pukyong National University, Busan 48513, Republic of Korea; vgawande77@gmail.com (V.G.);

**Keywords:** polyethylene, ultra-high molecular weight polypropylene, polymer blends, mechanical properties, rheological properties

## Abstract

The novel ultra-high molecular weight polypropylene (UHMWPP) as a dispersed component was melt blended with conventional high-density polyethylene (PE) and maleic anhydride grafted-polyethylene (mPE) in different proportions through a kneader. Ultra-high molecular weight polypropylene is a high-performance polymer material that has excellent mechanical properties and toughness compared to other polymers. Mechanical, thermal, and rheological properties were presented for various UHMWPP loadings, and correlations between mechanical and rheological properties were examined. Optimal comprehensive mechanical properties are achieved when the UHMWPP content reaches approximately 50 wt%, although the elongation properties do not match those of pure PE or mPE. However, it is worth noting that the elongation properties of these blends did not match those of PE or mPE. Particularly, for the PE/UHMWPP blends, a significant drop in tensile strength was observed as the UHMWPP content decreased (from 30.24 MPa for P50U50 to 13.12 MPa for P90U10). In contrast, the mPE/UHMWPP blends demonstrated only minimal changes in tensile strength (ranging from 29 MPa for mP50U50 to 24.64 MPa for mP90U10) as UHMWPP content varied. The storage modulus of the PE/UHMWPP blends increased drastically with the UHMWPP content due to the UHMWPP chain entanglements and rigidity. Additionally, we noted a substantial reduction in the melt index of the blend system when the UHMWPP content exceeded 10% by weight.

## 1. Introduction

Polyethylene (PE) is one of the most widely utilized thermoplastic materials globally, finding increasing applications in various industries. It has many advantages, including chemical resistance, electrical insulation, and low cost [1,2]. However, it also has some disadvantages, such as low mechanical strength and wear resistance. These disadvantages are due to the flexible molecular structure of PE, which has relatively low molecular weight and intermolecular entanglement [3]. These limitations have prevented PE from being used in some applications that require high mechanical strength. Polymer blending has garnered growing attention within both the scientific and industrial sectors. Investigating high-toughness polyethylene blends holds significant scientific importance. Blending allows polymer–polymer mixtures to represent a unique category of compounds [4,5]. The material properties of polymers combined in an extruder undergo changes dependent on the achieved homogenization. We can distinguish between homogeneous and heterogeneous polymer blends, with two-phase blends often providing advantages over single-phase ones [6]. Beyond the individual component properties, the morphology of the blends and the interactions between different polymers can be harnessed to influence the characteristics of the blend [7]. Over the past few decades, there have been numerous studies focusing on enhancing the toughness of PE using various polymers and elastomers [8,9,10,11]. While these investigations have yielded satisfactory toughening effects, researchers have also been concerned about the deterioration in tensile properties associated with these systems.

Ultra-high molecular weight polypropylene (UHMWPP) possesses a molecular weight exceeding 10^6^ g/mol, and its characteristics are influenced more by its microstructure than its molecular mass. UHMWPP is classified as a semi-crystalline polymer, featuring crystallites with a lamellar structure. This structure results from chain folding within the crystalline phase. In contrast, in the amorphous phase, the chains are interconnected through random entanglements rather than folding [12,13]. Similar to ultra-high molecular weight polyethylene (UHMWPE), UHMWPP has long, linear polymer chains and high molecular weights, and can be applied to improve the mechanical strength of PE by blending. Previous studies from our group demonstrated that the processability of the UHMWPP can be improved by blending it with low molecular weight and moderate molecular weight polypropylene (PP) [13,14]. Generally, blending polyethylene with polypropylene typically results in immiscible blends [15,16]. Their molecular structure is quite similar, but due to the differences in the arrangement of the carbon atoms in their chemical structures, they do not readily mix at a molecular level. In most cases, when PE and PP are blended, they tend to phase separate, forming distinct PE-rich and PP-rich domains within the material [17,18,19]. Graziano et al. introduced a study on the properties of PE-rich blends with PP as the minor phase using maleic anhydride grafted PE as a compatibilizer, and they stated that the effectiveness of MAPE in improving the compatibility and mechanical properties of PE/PP blends [20]. Corroler et al. explored partial wetting in ternary polymer blends, specifically in PE/PP/PS and PE/PP/PC systems, focusing on the impact of polyethylene viscosity on PS droplet formation at the PE/PP interface during annealing. The study quantitatively analyzes PS droplet growth and coverage using image analysis. It demonstrates that polyethylene viscosity plays a significant role in partial wetting when the interfacial driving force is weak, affecting PS droplet size and surface coverage [21]. Jordan et al. highlighted the role of interfacial adhesion strength, polymer content, and processing conditions, which most affect the properties of polyolefin blends [22]. Jose et al. conducted a commendable study on iPP/HDPE blends. Their investigation revealed a two-phase structure in the blend’s phase morphology, and they attributed the deduction in mechanical properties to the incompatibility of the blend [23]. This phase separation can lead to a two-phase or immiscible blend, where each polymer retains its individual properties to a significant extent [10,24]. While immiscible blends can have some advantages in certain applications, such as impact modification and toughness improvement, researchers often employ compatibilizers or other techniques to enhance the compatibility between PE and PP and create more homogenous blends. These efforts aim to overcome the inherent immiscibility of the two polymers and improve their overall performance when combined.

We hypothesized that blending PE with UHMWPP could lead to synchronous toughening and reinforcing effects. The mechanical properties of the PE/UHMWPP blend were significantly influenced by the immiscible blend morphology. Aiming to improve interfacial miscibility and intermolecular entanglements of the PE/UHMWPP system, in this work, the maleic anhydride grafted polyethylene (mPE) was used to blend with the UHMWPP. Thus, it was reasonable to anticipate that the combination of mPE and UHMWPP would provide the advantages of their individual components, based on the inherent polymer properties, to potentially achieve a synergistic effect on both mechanical and rheological properties. However, working with UHMWPP, given its ultra-high molecular weight, presented challenges due to its extremely high melt viscosity, rendering it unsuitable for conventional processing techniques [12,13,14,25]. To address this issue, we utilized a kneader for the fabrication of both PE/UHMWPP and mPE/UHMWPP blends and evaluated the mechanical properties by comparing them with those of corresponding blends. Furthermore, we delved into investigating the morphology, thermal characteristics, and rheological properties of these blends.

## 2. Experimental

### 2.1. Materials

UHMWPP with a viscosity average molecular weight (Mv) of 1.57 × 10^6^ g/mol, high-density PE, and maleic anhydride grafted PE (mPE) were provided by Korea Petrochemical Ind. Co., Ltd. (Seoul, Republic of Korea). The basic formulation for the PE/UHMWPP and mPE/UHMWPP blends is detailed in Table 1. Antioxidants, specifically 2,6-di-ter-butyl-4-hydroxytoluene (BHT) from Junsei, Japan, and Songnox 1010 from Honshu Chemical Industry Co., Ltd., Wakayama, Japan, were employed.

### 2.2. Preparation of UHMWPP/PP Blends

**Figure 1:**
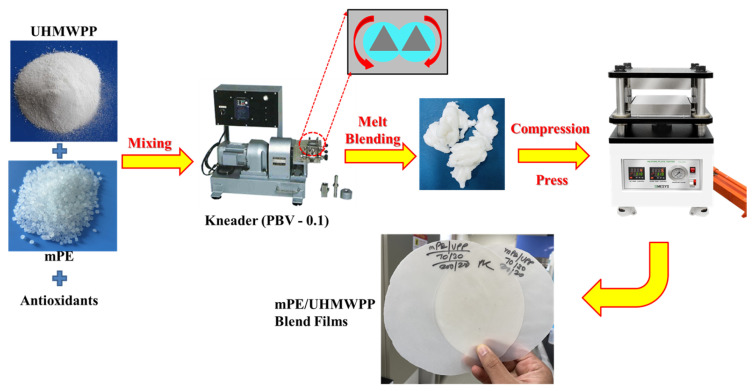
A schematic presentation of the preparation process of the PE/UHMWPP blends and mPE/UHMWPP blends.

PE, mPE, and UHMWPP were dehydrated in a vacuum oven at 60 °C to eliminate moisture before processing. Subsequently, they were pre-mixed with various compositions along with antioxidants, specifically BHT and Songnox 1010, both added at a concentration of 0.2 wt% of the polymer. Initially, different combinations of PE/UHMWPP and mPE/UHMWPP mixtures, along with antioxidants, were physically blended. Following this homogeneous pre-blending step, the mixtures were melted at 200 °C for 7 min using a kneader (PBV-0.1, Irie Shokai., Co., Ltd., Tokyo, Japan) operating at a screw speed of 50 rpm. PE, mPE, and blend films were then created using a compression press at a molding temperature of 200 °C under a pressure of 15–20 MPa for 10 min. Subsequently, they were allowed to cool to room temperature on a tabletop surface. The thickness of the resulting films was consistently maintained at 200–300 μm. A detailed depiction of the fabrication process is provided in Figure 1.

### 2.3. Characterizations

Mechanical properties of the pure polymers and blends were characterized by the Universal tensile machine. The Universal tensile machine (H1KT, Tinius Olsen, Horsham, PA, USA) was equipped with a 1 kN load sensor and operated at a stretching rate of 5 mm/min. For testing purposes, a minimum of six dog-bone-shaped replicas were cut from each blend film sample.

The thermal properties of the blends were assessed using differential scanning calorimetry (DSC, DSC 1, Mettler Toledo Inc., Zürich, Switzerland). The analysis involved heating up to 200 °C at a rate of 10 °C/min and cooling down to 0 °C at a rate of 300 °C/min under a nitrogen atmosphere. The % crystallinity Xc was determined using Equation (1).

Xc (%) = [(ΔH_m_/(ΔH_m_^100^ × W))] × 100 (1)

where ΔH_m_ (J/g) represents the melting enthalpy, W denotes the weight fraction of PE, mPE, or UHMWPP in the blends, and ΔH_m_100 is the melting enthalpy of the 100% crystalline PE (ΔH_m_^100^ = 281 J/g), 100% crystalline mPE ((ΔH_m_^100^ = 278 J/g), or UHMWPP (ΔH_m_^100^ = 207 J/g).

The rheological properties of the PE/UHMWPP and mPE/UHMWPP blend films were characterized using a melt index tester (MFI 9, AMETEK Lloyd Instruments Ltd., Horsham, PA, USA) with a standard test die. Tests were performed using both a 2.16 kg rod and a 21.6 kg rod at 230 °C.

Dynamic mechanical properties of the PE/UHMWPP and mPE/UHMWPP blend films were analyzed using an Ares G2 instrument (TA Instruments, New Castle, DE, USA). Measurements were carried out within a temperature range of −50 °C to 100 °C with a heating rate of 5 °C/min, while maintaining a constant frequency of 1.0 Hz.

Field emission scanning electron microscopy (FESEM, Tescan—MIRA3, Brno, Czech Republic) was used to observe the cross-sectional morphology of cryo-fractured surfaces of the PE/UHMWPP and mPE/UHMWPP blend films. The FESEM electron images were reported at an accelerating voltage of 3 kV for PE/UHMWPP and mPE/UHMWPP blend films.

## 3. Results and Discussions

### 3.1. Mechanical Properties

**Figure 2:**
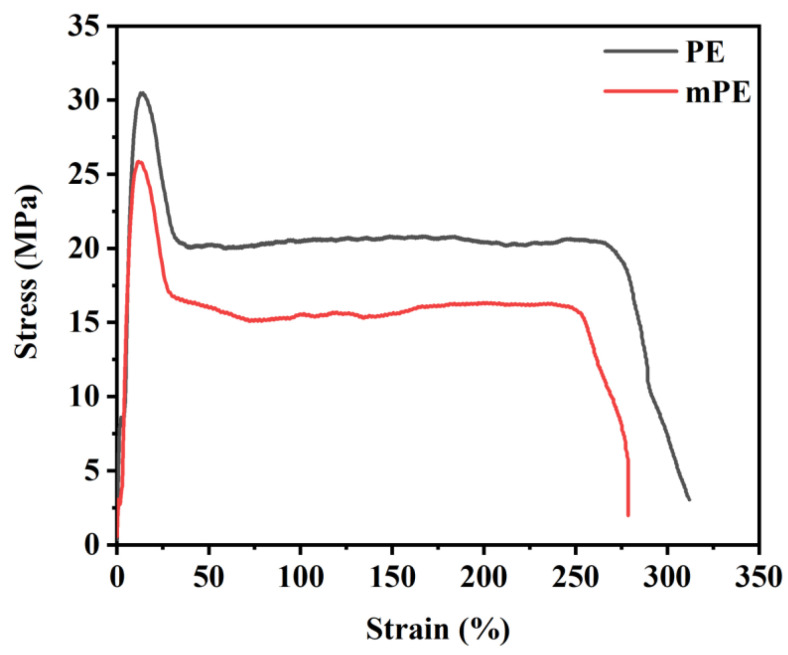
Examples of stress–strain behavior of PE and malleated PE (mPE) films.

Figure 2 illustrates the mechanical properties of the pure PE and mPE. Notably, it shows that the tensile strength of regular PE and mPE measures approximately 30.75 MPa and 25.10 MPa, respectively. As for elongation at break, PE and mPE exhibit values of around 329.77% and 273.33%, respectively. It is crucial to emphasize that the physical and mechanical properties of these polymers, including viscosity, diffusion rate, ability to be drawn, and toughness, are significantly impacted by both the molecular weight and its distribution [25,26,27]. The ultra-high molecular weight polypropylene (UHMWPP), due to its exceptionally high molecular weight and resulting high melt viscosity, faces challenges in melting adequately, even with increased processing temperatures. This difficulty in processing poses obstacles to its commercialization. Efforts were undertaken to produce films by blending UHMWPP with antioxidants; nevertheless, various challenges were encountered during the compounding and processing stages of the UHMWPP films.

**Figure 3:**
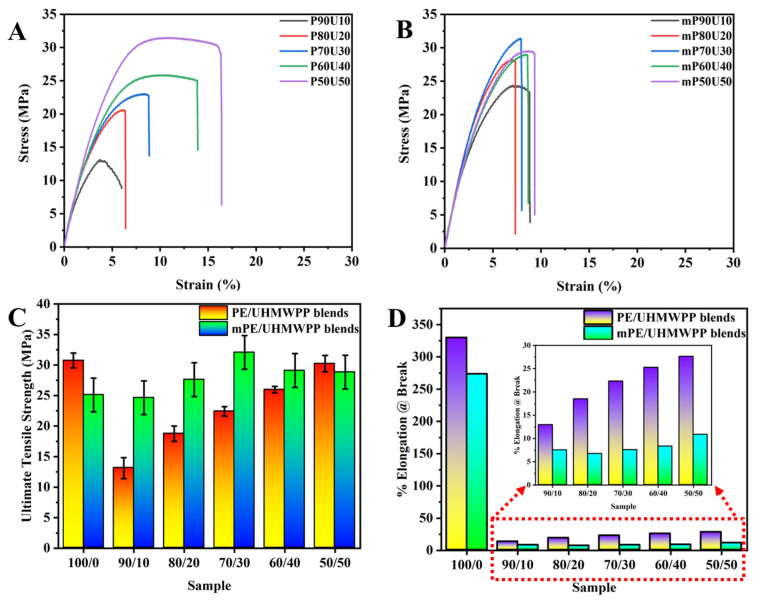
(**A**) Examples of stress–strain behavior of PE/UHMWPP blend and (**B**) mPE/UHMWPP blend films. (**C**) Ultimate tensile strength and (**D**) % elongation at break of PE/UHMWPP blend and mPE/UHMWPP blend films respective to its composition.

Figure 3 illustrates the mechanical properties of PE/UHMWPP and mPE/UHMWPP blends produced through a melt blending process. As depicted in Table 2, it is known that pure PE exhibits high tensile strength and elongation at break, whereas mPE’s performance in these aspects is comparatively modest. In these blends, an increase in UHMWPP content results in a significant decrease in elongation at break. Even with the addition of UHMWPP to mPE, there is no notable improvement in elongation at break. Figure 3 and Table 2 provide insights into the influence of UHMWPP content on the mechanical properties. It becomes evident that the mechanical properties improve as the amount of UHMWPP in the blends increases. Optimal comprehensive mechanical properties are achieved when the UHMWPP content reaches approximately 50 wt%, although the elongation properties do not match those of pure PE or mPE. In the case of PE/UHMWPP blends, when the UHMWPP content decreases to 40 wt%, the tensile strength decreases from 30.24 MPa to 25.97 MPa for the PE/UHMWPP_60/40_ blend. For mPE/UHMWPP blends, the tensile strength remains relatively stable at around 24.64 MPa, even with the addition of 10 wt% of UHMWPP. In contrast, the PE/UHMWPP_90/10_ blend sees a significant decrease in tensile strength to 13.12 MPa. The inclusion of mPE leads to a substantial enhancement in the tensile strength of the blends compared to PE/UHMWPP blends. In mPE/UHMWPP blends, as the UHMWPP content increases, the tensile strength gradually rises (except mPE/UHMWPP_50/50_ blend). Particularly, the addition of UHMWPP in mPE/UHMWPP blends within the 30–40% range exhibits favorable results. However, once the UHMWPP content surpasses 40 wt%, the tensile strength starts to decrease gradually.

**Figure 4:**
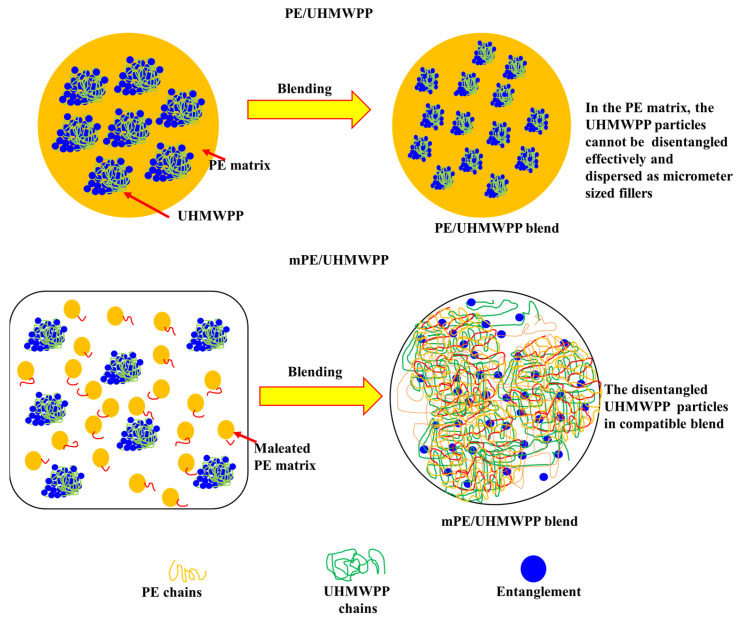
Schematic illustration of dual network for the PE/UHMWPP and mPE/UHMWPP blends.

As shown in Figure 4, for PE/UHMWPP, the UHMWPP phase encounters significant challenges in disentangling effectively during the melt blending process. The difficulty in effectively disentangling the UHMWPP phase during the melt blending process can be attributed to the shearing of the melt. UHMWPP has extremely long polymer chains, resulting in a high degree of entanglement in its natural state. When subjected to the shear forces and elevated temperatures involved in the melt blending process, the long polymer chains become entangled or agglomerated, making it challenging to achieve a uniform dispersion or disentanglement. This results in its dispersion as micrometer-sized fillers within the PE matrix. The primary reason behind this limited dispersal is the substantial viscosity mismatch between the two phases [2,3,28]. Consequently, this leads to poor compatibility and consequently results in the blends exhibiting relatively low mechanical properties. In contrast, mPE/UHMWPP blends feature mPE, which is a modified variant of PE chemically grafted with maleic anhydride. This modification imparts enhanced compatibility between mPE and UHMWPP, facilitating improved interfacial covalent linkage between the two phases within the blend system. Furthermore, the molecular chain interdiffusion is notably enhanced in this scenario. As a result, the UHMWPP phase experiences dissolution and becomes a compatible dual network structure. This structural transformation enhances the overall performance of the blend by promoting stronger interactions between its constituents than the PE/UHMWPP blends. The presence of mPE in the blend improves the compatibility of mPE and UHMWPP, leading to a more uniform distribution of the polymers and potentially enhancing the overall properties and performance of the blend than PE/UHMWPP blends.

### 3.2. Thermal Properties

**Figure 5:**
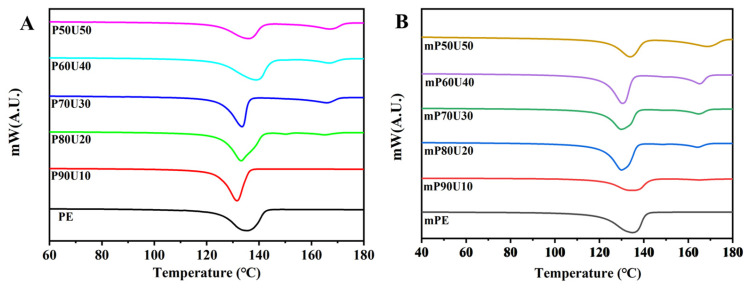
DSC thermograms of (**A**) PE/UHMWPP and (**B**) mPE/UHMWPP blend films.

Figure 5 presents the thermal properties of the PE, mPE, and their binary blend as analyzed by DSC. The corresponding data for melting temperature and % crystallinity are summarized in Table 2. In the DSC curves shown in Figure 5, both PE and mPE exhibit single melting temperatures (T_m_) of approximately 135.3 °C and 135.5 °C, respectively. Notably, a distinct T_m_ at approximately 166 ± 3 °C corresponds to UHMWPP, while another T_m_ at about 133 ± 4 °C is attributed to PE and mPE.

Upon the addition of UHMWPP to the blend, a noteworthy change is observed compared to the curves of the pure polymers. Blends containing more than 10 wt% UHMWPP display two distinct melting peaks, aligning with the respective T_m_ values of the PE and UHMWPP phases in the blends. Interestingly, the T_m_ of the PE phase appears to be minimally affected by the inclusion of UHMWPP, indicating the migration of nuclei from one phase to another. The migration of nuclei, which are small crystalline or ordered regions within the polymer, can occur in some polymer blends. This migration can affect the overall T_m_ of the blend because the crystalline regions from one phase may influence the crystallization behavior of the other phase. This phenomenon can lead to a shift in the T_m_ or changes in the melting behavior of the blend. In this case, the observation that the T_m_ of the PE phase is minimally affected by the inclusion of UHMWPP suggests that there is limited interaction or compatibility between PE and UHMWPP. In many cases, blend introduces a secondary melting peak at a higher temperature, indicating the presence of crystalline regions within the blend. The secondary T_m_ suggests that the blends may have a complex crystalline structure due to the coexistence of PE or mPE and UHMWPP. Furthermore, the crystallinity of the blends also varies with composition. The blend composition, specifically the ratio of PE (or mPE) to UHMWPP, plays a significant role in determining the overall crystallinity. As the UHMWPP content increases, it can disrupt the crystalline structure of the PE or mPE, leading to a decrease in their crystallinity. In contrast, UHMWPP itself may have a distinct crystalline structure that contributes to the increased crystallinity of the UHMWPP component in the blends. The PE/UHMWPP blends exhibit a crystallinity of around 72–75% (PE crystalline %) and 24–28% (UHMWPP crystallinity %), while the mPE/UHMWPP blends maintain a crystallinity of around 62–64% (mPE crystalline %) and 18–35 (UHMWPP crystallinity %). This is because the maleic anhydride groups in mPE interfere with the crystallization of PE [28,29,30]. The thermal properties of the blends have a significant impact on their mechanical performance. The mPE/UHMWPP blends demonstrate higher tensile strength and lower elongation than the PE/UHMWPP blends. This enhancement can be attributed to the maleic anhydride groups in mPE, which promotes the formation of stronger interfacial bonds between the mPE and UHMWPP phases. Overall, the thermal properties of the PE/UHMWPP and mPE/UHMWPP blends can be tailored by adjusting the composition of the blend. The mPE/UHMWPP blends offer superior mechanical performance than PE/UHMWPP blends, making them suitable for a wider range of applications.

### 3.3. Rheological Properties

**Figure 6:**
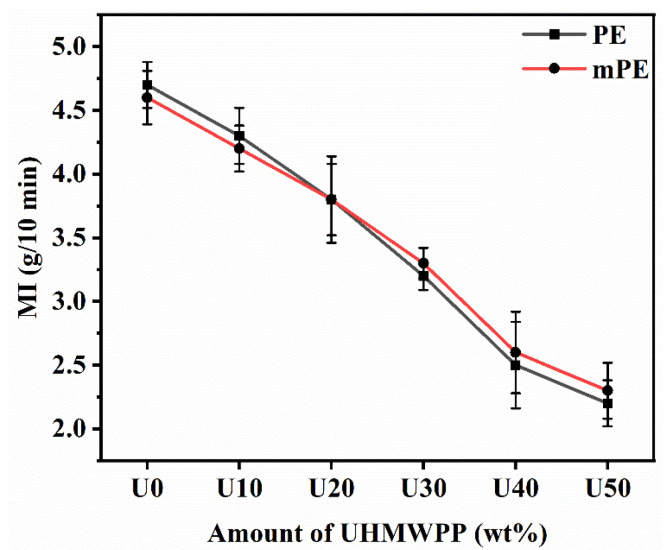
Melt index of PE/UHMWPP and mPE/UHMWPP blend films (2.16 kg rod, 230 °C, average of six times measurement).

The melt index (MI) of a polymer material is known to be inversely related to the molecular weight and melt viscosity of the polymer [29]. In order to assess the impact of UHMWPP content on the melt flow characteristics, the MI of both PE/UHMWPP and mPE/UHMWPP blends was analyzed, as illustrated in Figure 6. The melt index (MI) of the PE/UHMWPP and mPE/UHMWPP blends decreases as the amount of UHMWPP increases. This is because UHMWPP has a higher molecular weight than PE, and higher molecular weight polymers have lower melt indices. The melt viscosity of the UHMWPP was very high since the observed MI was only 0.87 g/10 min after using a 21.6 kg rod at 230 °C. The MI of the mPE/UHMWPP blends is quite similar to that of the PE/UHMWPP blends at all UHMWPP contents. Generally, maleic anhydride groups in mPE promote the formation of intermolecular entanglements, and miscible blends typically have lower melt indices than immiscible blends. MI is influenced by the viscosity of the polymer. The presence of maleic anhydride groups in mPE may alter its rheological properties compared to pure PE. Viscosity can be influenced by molecular weight, branching, and the degree of entanglement in the polymer chains. The extent of these changes may result in different MI values [31]. The decrease in MI with increasing UHMWPP content is more pronounced for the mPE/UHMWPP and PE/UHMWPP blends compared to the pure PE and mPE.

**Figure 7:**
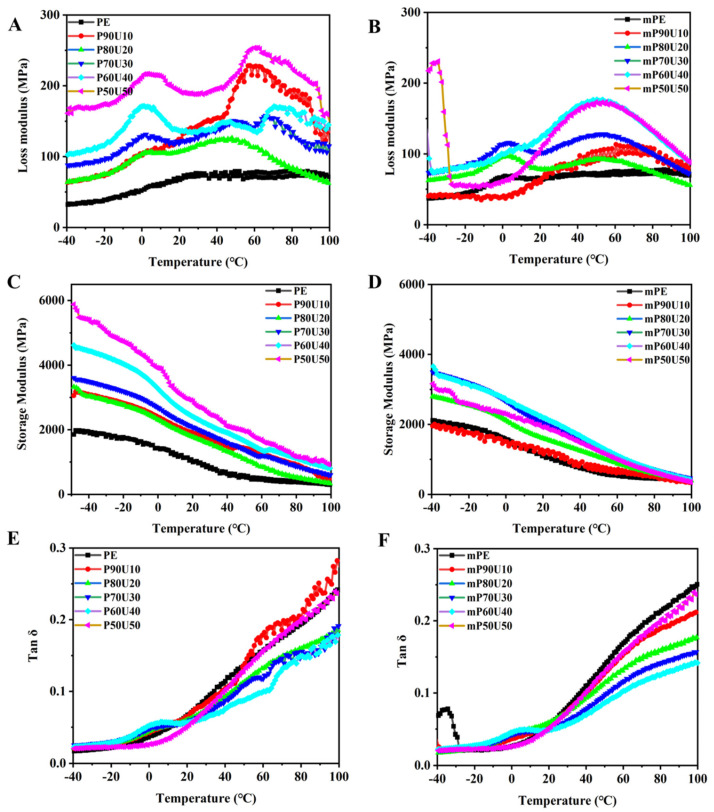
The dynamic (**A**,**B**) Loss modulus, (**C**,**D**) storage modulus, and (**E**,**F**) tan δ of PE/UHMWPP and mPE/UHMWPP blend films.

Dynamic rheological measurements were conducted on the PE/UHMWPP and mPE/UHMWPP blends, and the results are depicted in Figure 7. In general, the melt rheological behavior of these polymer blends is closely linked to factors such as molecular weight, viscosity ratio, component compatibility, and even the phase structure within the blends [32,33]. Figure 7A,B illustrate the variations in loss modulus with temperature for the PE/UHMWPP and mPE/UHMWPP blends, revealing the corresponding relaxation processes. The loss modulus signifies the viscous characteristics of the material, indicating its ability to dissipate energy. Conversely, the storage modulus represents the material’s elasticity and stiffness. Figure 7A–D provide insight into the changes in the loss modulus and storage modulus with temperature for both the PE/UHMWPP and mPE/UHMWPP blends, highlighting their respective relaxation behaviors. Notably, the PE/UHMWPP blends exhibit higher storage and loss moduli compared to pure PE. This phenomenon can be attributed to the interface between the PE and UHMWPP phases, which act as a reinforcing mechanism. At this interface, polymer chains restrict the movement of chains in the bulk material, resulting in increased storage and loss moduli. Additionally, due to the very high molecular weight of the UHMWPP, it may have higher storage and loss modulus than PE and mPE. The storage modulus of the PE/UHMWPP blends exhibited a significant increase with higher UHMWPP content. This can be attributed to the formation of chain entanglements and increased rigidity in the UHMWPP component, which possesses a considerably longer relaxation time compared to the PE chain due to its high molecular weight. UHMWPP has significantly longer polymer chains than conventional PP, which means the polymer chains can become more entangled or knotted in the bulk of the material. These entanglements result in an increase in the material’s rigidity and longer relaxation times. This is a property that contributes to the outstanding strength and toughness of UHMWPP. Regarding the interface between UHMWPP and PE, these two polymers typically have poor adhesion due to differences in their chemical structures and polarities. At the interface, there may be limited intermolecular interactions or adhesion between the two phases. The observed chain entanglements and increased rigidity in UHMWPP mainly affect the UHMWPP phase itself and its bulk properties. Notably, Tan δ has proven to be more responsive to network formation than the modulus. The tan δ value of both PE/UHMWPP and mPE/UHMWPP blends decrease as the temperature increases (as shown in Figure 7E,F). With an increasing proportion of UHMWPP, the tan δ values of the mPE/UHMWPP blends dramatically decreased, indicating enhanced toughness in blends. However, the tan δ of the mPE/UHMWPP blends is lower than the tan δ of the PE/PP blends at all temperatures. This is because the maleic anhydride groups in mPE interfere with the crystallization of PE, and less crystalline polymers have broader and lower tan δ peaks, consistent with the findings from DSC analysis.

### 3.4. Morphology of Blends

**Figure 8:**
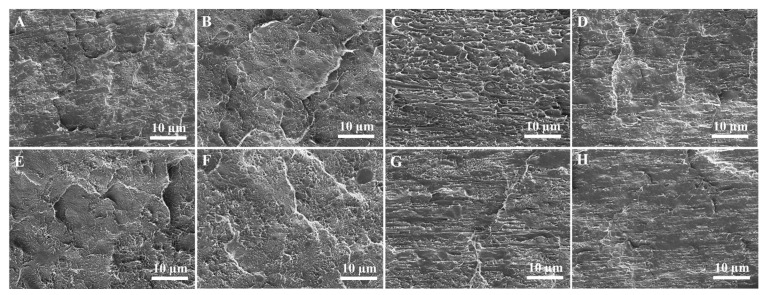
SEM micrographs of the cyro-fractured surfaces of (**A**) PE, (**B**) P90U10, (**C**) P70U30, (**D**) P50U50, (**E**) mPE, (**F**) mPE90U10, (**G**) mPE70U30, and (**H**) mPE50U50 blend samples.

Figure 8 shows the SEM micrographs of the cryogenically fractured surfaces of both the PE/UHMWPP and mPE/UHMWPP blends. In the case of the PE micrographs, we observe a relatively smooth surface characterized by small pores and voids, typical of semi-crystalline polymers and indicative of a relatively ductile material. Shifting our attention to the SEM micrograph of the mPE sample, we note a surface quite similar to the PE sample, albeit with a slightly greater number of voids. This is likely due to the presence of the maleic anhydride groups, which can introduce some branching and crosslinking into the PE chains. However, the P90U10 micrograph also shows some evidence of phase separation, with small dark regions dispersed in a lighter matrix. This pattern points towards the presence of UHMWPP particles in the blend. However, due to the limited flowability of UHMWPP, the dispersion of UHMWPP in PP appears uneven, concluding in phase separation between PE and UHMWPP. As a result, it does not form a well-connected structure with PE, and this lack of interconnection leads to low mechanical properties in the blend. Moving forward, the SEM micrographs of the P70U30 and P50U50 samples unveil a more pronounced phase separation between the PE and UHMWPP phases. The PE phase emerges as a continuous matrix, with the UHMWPP phase scattered throughout the matrix as distinct particles. A similar particle dispersion morphology was observed for PE/PP blends [28,34]. These UHMWPP particles are notably larger, and evidence of interfacial debonding between the two phases comes to the fore.

The SEM micrograph of the mPE90U10 sample shows a similar morphology to the P90U10 sample, but with a slightly greater number of voids. This is likely due to the presence of the maleic anhydride groups, which can reduce the interfacial tension between the PE and UHMWPP phases, leading to a finer dispersion of the UHMWPP phase [35]. The SEM micrographs of the mPE50U50 samples show a similar morphology to the P50U50 sample, but with a slightly finer dispersion of the PE and UHMWPP phases. This is likely due to the presence of the maleic anhydride groups, which can improve the compatibility between the PE and UHMWPP phases. Additionally, we observe a multitude of broken fibrils attributable to the pulled-out UHMWPP phase, demonstrating that strong interfacial interaction between the two significantly improved the blend compatibility.

The mPE/UHMWPP blends may be brittle compared to pure PE or mPE, limiting flexibility. However, the potential applications for such blends may include scenarios where extreme rigidity and tensile strength are more critical than flexibility, such as in structural components or load-bearing elements. Further research and development may uncover specific niche applications that can benefit from the characteristics of the blends.

## 4. Conclusions

In this study, we investigated the effects of UHMWPP on the mechanical, thermal, and rheological properties of the PE/UHMWPP blends, and these blends were fabricated using a melt blending technique by using a kneader. The novel UHMWPP is a high-performance polymer material that has excellent mechanical properties and toughness compared to other polymers. However, the inclusion of UHMWPP into PE and mPE led to a reduction in processing capabilities due to a decrease in the melt index (MI) as UHMWPP content increased. The addition of UHMWPE in PP did not improve the mechanical properties due to the poor compatibility between UHMWPP and PE. Entanglements of UHMWPP chains were found to influence the tensile strength of the blends. The incorporation of the UHMWPP to mPE produces a rigid amorphous phase, which results in enhancement in tensile strength, although the % crystallinity is reduced. With increasing UHMWPP content, the tan δ values of the mPE/UHMWPP blends dramatically decreased, indicating the increasing toughness of the blends. The storage modulus of the PE/UHMWPP blends were increased drastically with the UHMWPP content due to the UHMWPP chain entanglements and rigidity, which have a comparatively much longer relaxation time than the PE chain due to the high molecular weight of UHMWPP. The loss modulus, representing dissipative energy, was higher for the blends compared to pure polymers.

## Figures and Tables

**Table 1:** Basic formulation to prepare PE/UHMWPP blends and mPE/UHMWPP blends.

Sample Designation	PE (wt%)	mPE (wt%)	UHMWPP (wt%)
PE	100	-	0
P90U10	90	-	10
P80U20	80	-	20
P70U30	70	-	30
P60U40	60	-	40
P50U50	50	-	50
mPE	-	100	0
mP90U10	-	90	10
mP80U20	-	80	20
mP70U30	-	70	30
mP60U40	-	60	40
mP50U50	-	50	50

**Table 2:** Mechanical and thermal properties of PE, mPE, PE/UHMWPP blends, and mPE/UHMWPP blend films.

Sample	Ultimate Tensile Strength (MPa)	Elongation @ Break	T_m_ (°C)	Crystallinity (%)
PE	30.75 ± 1.21	329.77 ± 16.82	135.3	76.28
P90U10	13.12 ± 1.72	12.93± 1.55	131.6	72, -
P80U20	18.76 ± 1.26	18.49 ±1.22	133.1, 165	72.28, 24.73
P70U30	22.41 ± 0.78	22.31 ± 0.80	133.6, 166.6	75.29, 25.50
P60U40	25.97 ± 0.53	25.28 ± 0.60	137, 167	68.33, 27.49
P50U50	30.24 ± 1.33	27.65 ± 1.3	136, 167.6	62.28, 24
mPE	25.10 ± 1.53	273.33 ± 27.76	135.5	67.80
mP90U10	24.64 ± 0.93	7.48 ± 1.11	134.3, 165.8	65.38, 18.21
mP80U20	27.60 ± 0.74	6.70 ± 0.35	131, 164.3	63.93, 27.68
mP70U30	32.07 ± 0.75	7.54 ± 0.46	131.3, 164.6	62.10, 28.36
mP60U40	29.10 ± 0.73	8.31 ± 0.96	131.4, 165.3	63.12, 35.25
mP50U50	28.83 ± 0.85	10.79 ± 0.40	133.8, 168.6	56.70, 28.50

## Data Availability

Not applicable.
